# Night Temperature Affects the Growth, Metabolism, and Photosynthetic Gene Expression in *Astragalus membranaceus* and *Codonopsis lanceolata* Plug Seedlings

**DOI:** 10.3390/plants8100407

**Published:** 2019-10-10

**Authors:** Ya Liu, Xiuxia Ren, Byoung Ryong Jeong

**Affiliations:** 1 Department of Horticulture, Division of Applied Life Science (BK21 Plus Program), Graduate School of Gyeongsang National University, Jinju 52828, Korea; liuya113@mails.ucas.ac.cn (Y.L.); renxiuxia@caas.cn (X.R.); 2 Institute of Agriculture and Life Science, Gyeongsang National University, Jinju 52828, Korea; 3 Research Institute of Life Science, Gyeongsang National University, Jinju 52828, Korea

**Keywords:** *GBSS*, growth, *FDX*, medicinal plant, night temperature, stomata, *RBCL*, total phenols and flavonoids

## Abstract

*Astragalus membranaceus* and *Codonopsis lanceolata* are two important medical herbs used in traditional Oriental medicine for preventing cancer, obesity, and inflammation. Night temperature is an important factor that influences the plug seedling quality. However, little research has focused on how the night temperature affects the growth and development of plug seedlings of these two medicinal species. In this study, uniform plug seedlings were cultivated in three environmentally controlled chambers for four weeks under three sets of day/night temperatures (25/10 °C, 25/15 °C, or 25/20 °C), the same relative humidity (75%), photoperiod (12 h), and light intensity (150 μmol·m^−2^·s^−1^ PPFD) provided by white LEDs. The results showed that night temperature had a marked influence on the growth and development of both species. The night temperature of 15 °C notably enhanced the quality of plug seedlings evidenced by the increased shoot, root, and leaf dry weights, stem diameter, and Dickson’s quality index. Moreover, a night temperature of 15 °C also stimulated and increased contents of primary and secondary metabolites, including soluble sugar, starch, total phenols and flavonoids. Furthermore, the 15 °C night temperature increased the chlorophyll content and stomatal conductance and decreased the hydrogen peroxide content. Analysis of the gene expression showed that *granule-bound starch synthase* (*GBSS*), *ribulose bisphosphate carboxylase large chain* (*RBCL*), and *ferredoxin* (*FDX*) were up-regulated when the night temperature was 15 °C. Taken together, the results suggested that 15 °C is the optimal night temperature for the growth and development of plug seedlings of *A. membranaceus* and *C. lanceolata*.

## 1. Introduction

As two important medicinal herbs in Oriental medicine, *Astragalus membranaceus* and *Codonopsis lanceolata* are mainly distributed in East Asia, especially in Korea, China, and Japan [1,2]. It has been widely reported that the roots of these two species can be used for preventing diseases including inflammation, obesity, and cancer [3,4,5]. In addition, as a high-class vegetable, *C. lanceolata* has been consumed as food for several centuries, especially in ancient Korea [6,7]. Nowadays, new medicinal compounds continue to be reported from these two species, and their medicinal mechanisms have been demonstrating [3,8,9]. Focused research on the separation, extraction, and characteristics of the phytochemicals of these two species are ongoing [8,10]. However, there has been little work done on the cultivation of these two species in an environmentally controlled conditions for obtaining high quality plug seedlings.

Temperature is an important abiotic factor that has a great influence on the growth, yield, and quality of crop plants and shapes the morphology of seedlings [11,12,13]. High and low temperature extremes usually contribute to physiological disorders and dysfunction, resulting in a loss of yield and a degradation of quality [14]. Previous studies widely report on such effects in a variety of species, including rice [15], wheat [16], tomato [17], strawberry [18], and lettuce [19]. In recent years, more and more researchers have paid attention to the effects of the night temperature on crops [20,21,22,23]. It seemed that low and high night temperatures caused a reduction in the biomass with different patterns. For example, Jing et al. [24] found that the effects of a high night temperature were primarily related to biomass allocation to seeds, while the effects of a low night temperature were more connected with the total biomass. The results from Loka and Oosterhuis [25] showed that two high temperature regimes (27 °C and 30 °C) caused a significant increase in the respiration rate (by 49% and 56% respectively), compared to the control (24 °C). However, an opposite result has also been reported. Kanno et al. [26] found that the final biomass of rice (*Oryza sativa* L.) was the greatest in plants grown at the highest night temperature (27 °C) rather than in those grown with lower night temperatures (17 °C and 22 °C). Moreover, there is little research regarding the effects of the night temperature on the growth and development of plug seedlings. Therefore, further research is still needed to clarify how the night temperature influences the growth, biomass, and quality, especially in plug seedlings of medicinal plants.

Photosynthesis is an important physiological process that is strictly regulated by a series of enzymes [27]. In light–dependent reactions, ferredoxin, coded by the *FDX* gene, is an important iron–sulfur protein that accepts electrons produced from sunlight-excited chlorophyll and mediates them to NADP^+^ in the electron transport chain [28]. In dark reactions, ribulose bisphosphate carboxylase (Rubisco, coded by the *RBC* gene) is a crucial enzyme in the initial step of the Calvin cycle in plants, since it catalyzes the primary CO_2_ fixation step and controls the photosynthesis rate [29]. Additionally, granule-bound starch synthase, coded by the *GBSS* gene, is indispensable for the synthesis of linear glucan (amylose) in starch and also takes part in building the final structure of amylopectin [30,31,32]. Previous studies have proven that those enzymes are sensitive to the temperature. The expression of the *FDX*, *GBSS*, and *RBC* genes are suppressed by high or low day temperatures, resulting in a reduction of crop yield [33,34,35]. However, few studies have focused on how those genes respond to the night temperature.

In the current study, it was hypothesized that a suitable night temperature (15 °C) could enhance the plug seedling quality and too high or low temperatures would have a negative effect on the growth and development of *A. membranaceus* and *C. lanceolata* plug seedlings. In order to test the above hypothesis, uniform plug seedlings were cultivated at night temperatures of 10 °C, 15 °C, and 20 °C for four weeks. Characteristics of the growth, development and morphology of *A. membranaceus* and *C. lanceolata* plug seedlings grown with different night temperatures were evaluated. The contents of the primary and secondary metabolites such as soluble sugar, starch, total phenols, and flavonoids, were further determined. Moreover, photosynthesis-related parameters including the chlorophyll content and stomatal conductance were measured. In addition, the expression of *FDX*, *RBC*, and *GBSS* genes were assayed. These data could provide a theoretical and practical basis not only for enhancing the quality of plug seedlings by manipulating the night temperature, but also for guarding the management of other medicinal plants.

## 2. Results

### 2.1. Growth, Development and Morphology

**Figure 1:**
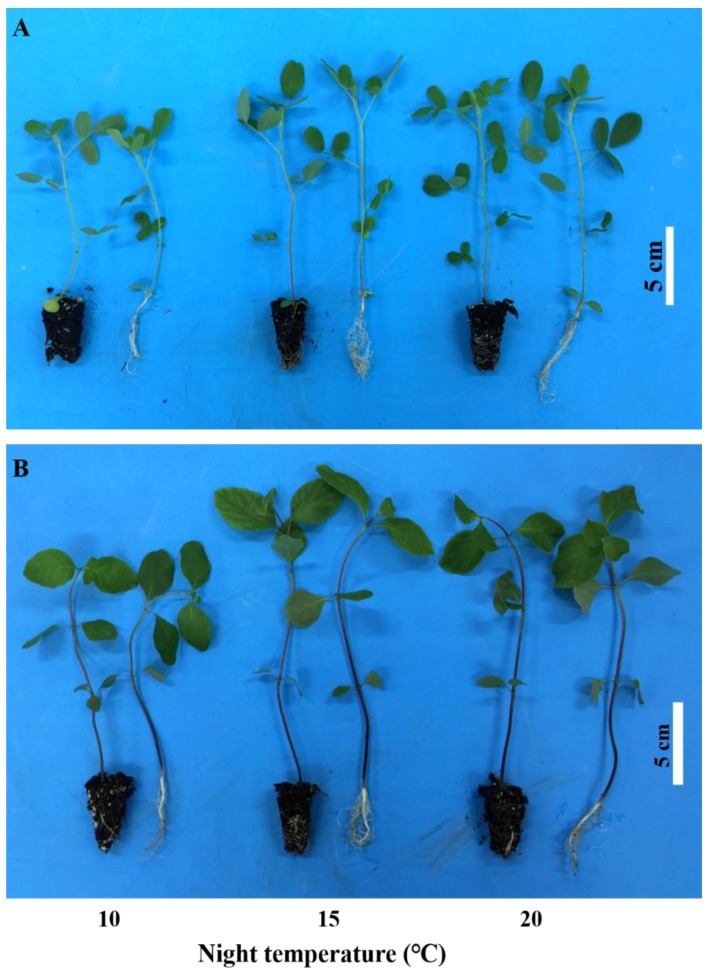
Morphological characteristics of plug seedlings of *Astragalus membranaceus* (**A**) and *Codonopsis lanceolata* (**B**) as affected by the night temperature. Bar, 5 cm.

In this study, the night temperature had a remarkable influence on the growth, development, and morphology of plug seedlings. As shown in Figure 1, a diverse morphology of plug seedlings of *A. membranaceus* and *C. lanceolata* were shaped by different night temperatures. Compared with 15 °C and 20 °C, a night temperature of 10 °C markedly inhibited the growth and development of both species with dwarfed shoots, curved stems, and sparse roots. Plug seedlings grew better with a night temperature of 15 °C than with 20 °C, evidenced by the slightly higher biomass, stem diameter, and Dickson’s quality index (DQI), although differences in the growth parameters of seedlings between these two treatments were not significant (Table 1 and Table 2).

### 2.2. Contents of Soluble Sugar and Starch

**Figure 2:**
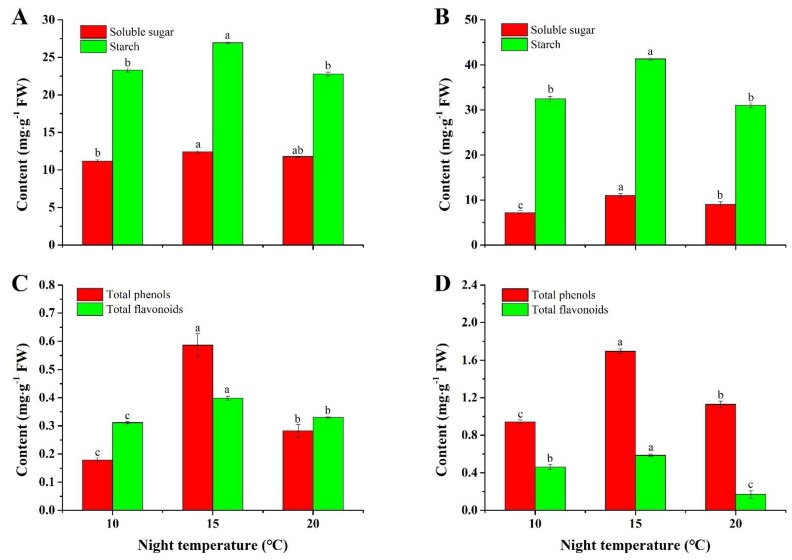
Contents of soluble sugar, starch, total phenols, and flavonoids in *A. membranaceus* (**A**,**C**) and *C. lanceolata* (**B**,**D**) plug seedlings as affected by night temperature. Data are presented as the mean ± standard error (n = 3). Different letters (a, b, and c) indicate significant differences among treatments by Duncan’s multiple range test at a 0.05 level.

In this research, the night temperature had an impact on the primary metabolism in *A. membranaceus* and *C. lanceolata* plug seedlings (Figure 2A,B). The same feature was found in both species where the contents of soluble sugar and starch were increased first and then decreased as the night temperature was raised from 10 °C to 20 °C. The maximum contents of soluble sugar and starch were 12.4 ± 0.2 and 27.0 ± 0.1 mg·g^−1^ FW for *A. membranaceus*, and 11.0 ± 0.4 and 41.3 ± 0.3 mg·g^−1^ FW for *C. lanceolata*, respectively.

### 2.3. Contents of Total Phenols and Flavonoids

The data showed that secondary metabolites were markedly influenced by different night temperatures (Figure 2C,D). In *A. membranaceus,* maximum contents of both total phenols and flavonoids were observed when grown at 15 °C, where they were respectively 0.59 ± 0.04 and 0.40 ± 0.01 mg·g^−1^ FW. The highest contents of total phenol and flavonoid were observed at 15 °C, followed by those at 20 °C and 10 °C. In *C. lanceolata*, the contents of total phenols and flavonoids were 1.70 ± 0.03 and 0.59 ± 0.01 mg·g^−1^ FW at 15 °C, significantly greater than those grown with the other two night temperatures.

### 2.4. Hydrogen Peroxide Content

**Figure 3:**
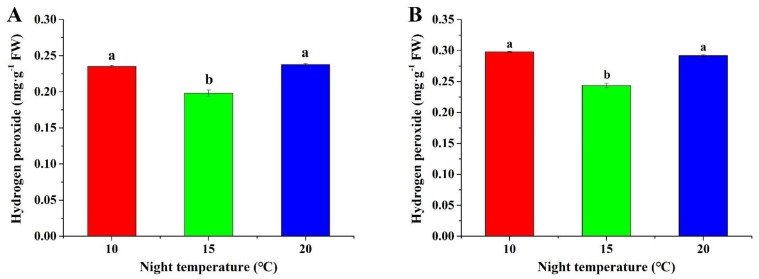
Effect of night temperature on the hydrogen peroxide content in *A. membranaceus* (**A**) and *C. lanceolata* (**B**) plug seedlings. Data are presented as the mean ± standard error (n = 3). Different letters (a and b) indicate significant differences among treatments by Duncan’s multiple range test at a 0.05 level.

As shown in Figure 3, the lowest H_2_O_2_ content in *A. membranaceus* and *C. lanceolata* was found at 15 °C, which were respectively 0.198 ± 0.004 and 0.244 ± 0.004 mg·g^−1^ FW. A lower or higher night temperature (10 °C and 20 °C) significantly increased the H_2_O_2_ content.

### 2.5. Chlorophyll Content and Stomatal Conductance

**Figure 4:**
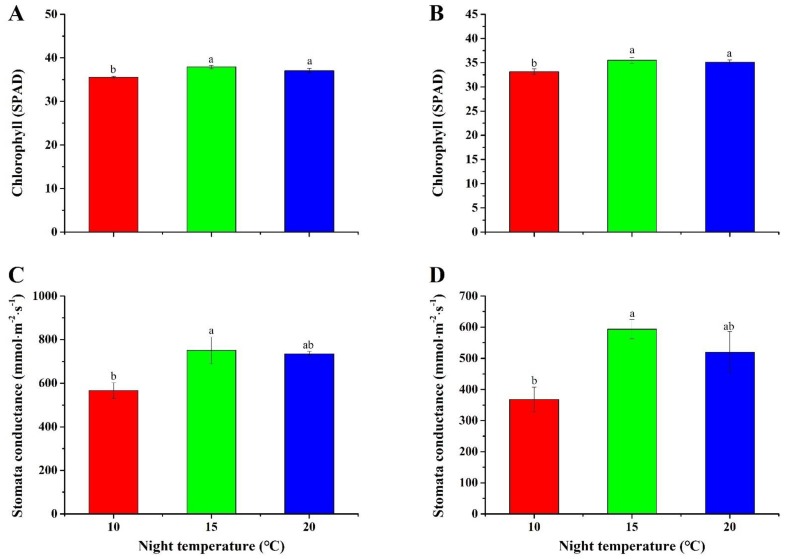
Effect of night temperature on the chlorophyll content and stomatal conductance in plug seedlings of *A. membranaceus* (**A**,**C**) and *C. lanceolata* (**B**,**D**). Data are presented as the mean ± standard error (n = 3). Different letters (a and b) indicate significant differences among treatments by Duncan’s multiple range test at a 0.05 level.

In this study, an elevated night temperature (15 °C) increased the chlorophyll content in plug seedlings of both *A. membranaceus* and *C. lanceolata* (Figure 4A,B). Slightly higher chlorophyll content was observed in seedlings grown with a 15 °C night temperature, compared to those grown with a 20 °C night temperature, but the difference was not significant. 

In the current study, the stomatal conductance significantly increased with the increase of the night temperature from 10 °C to 15 °C, and then decreased slowly (Figure 4C,D). The peak values of the stomatal conductance were 749.7 ± 61.6 and 593.3 ± 31.0 mmol·m^−2^s^−1^ in plug seedling of *A. membranaceus* and *C. lanceolata*, respectively.

### 2.6. Gene Expression Analysis

**Figure 5:**
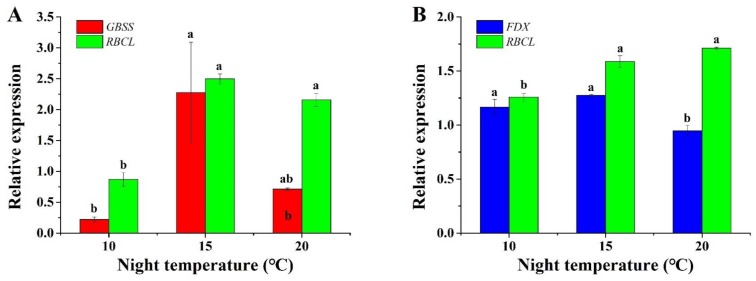
Effect of night temperature on the gene expression in *A. membranaceus* (**A**) and *C. lanceolata* (**B**) plug seedlings. *GBSS: granule-bound starch synthase, RBCL: ribulose bisphosphate carboxylase large chain*, and *FDX: ferredoxin*. The *18S* and *β-Actin* were used as the housekeeping genes in *A. membranaceus* and *C. lanceolata*, respectively. Data are presented as the mean ± standard error (n = 3). Different letters (a and b) indicate significant differences among treatments by Duncan’s multiple range test at a 0.05 level.

The data showed that expression of the photosynthetic genes was influenced by the night temperature (Figure 5). In *A. membranaceus*, a night temperature of 15 °C significantly increased the expression of *granule-bound starch synthase* (*GBSS*), and *ribulose bisphosphate carboxylase large chain* (*RBCL*), more than those at a night temperature of 10 °C. Although there were no significant differences between those at 15 °C and 20 °C, the expression of these two genes was higher at 15 °C than at 20 °C. Similarly, the highest expression of *ferredoxin* (*FDX*) was also at 15 °C in *C. lanceolata*. The expression of *RBCL* in *C*. *lanceolata* was remarkably greater at 15 °C and 20 °C than that at 10 °C. However, the *RBCL* expression at night temperatures of 15 °C and 20 °C was not significantly different.

## 3. Discussion

Temperature is one of crucial abiotic factors that greatly decides the quality and yield of crops [15,36]. Recently, an increasing number of studies have shown that the night temperature has a considerable influence on the growth, development, and morphology of plants [37,38,39]. In this study, a night temperature at 15 °C markedly improved the quality of *A. membranaceus* and *C. lanceolata* plug seedlings, evidenced by the higher biomass, stem diameter, and DQI. These results are supported by other studies. For instance, Jing et al. [24] found that high and low night temperatures both had a negative effect on the plant yield by collecting and analyzing data from 112 papers. Morita et al. [40] reported that the rate of grain growth in rice (*O. sativa* L.) was lower with a low night temperature than with a high night temperature. In addition, Cheng et al. [41] found that a high night temperature not only decreased the rice yield but also reduced the stimulatory effect of elevated CO_2_ levels on the rice production. However, an opposite conclusion in rice had been reported from Kanno et al. [26], who found that a high night temperature stimulated leaf photosynthesis and increased biomass production by promoting the growth of leaf blades. More research is needed to clarify this discrepancy and the underlying mechanisms regarding the same species.

The night temperature had an influence on the primary and secondary metabolism [20,42]. In the current study, a night temperature of 15 °C had a positive effect on the biosynthesis and accumulation of soluble sugar and starch in both species. Similar results have been reported by Loka and Oosterhuis [43], who found that high night temperatures significantly decreased carbohydrate concentrations such as hexose, sucrose, and starch in cotton (*Gossypium hirsutum* L.). The reason is that a high night temperature contributed to a boosted respiration, leading to an increased consumption of carbohydrates [25,44,45]. Another reason is due to the increased mobilisation and faster metabolic use of soluble sugar and starch in plug seedlings grown at 15 °C night temperature. Moreover, higher metabolic rates at 15 °C than 10 °C during the night will lead to a better preparation for the coming day, which partly explained the differences in quality of plug seedlings. Further study should be carried out to demonstrate how night temperature influences carbohydrates allocation, partition, and metabolic use. Besides, the data in this study showed that contents of total phenols and flavonoids were greatly increased by the 15 °C night temperature in *A. membranaceus* and *C. lanceolata*. Similarly, Ryu et al. [46] also found that the anthocyanin biosynthesis in apple was inhibited by a high night temperature. Li et al. [47] reported that phenolic compound accumulation in pitaya fruits increased with elevated temperature from 5 °C to 15 °C. However, Gaiotti et al. [22] found that the capacity of anthocyanin biosynthesis was increased by cool nights (10 °C–11 °C) during veraison of *Vitis vinifera*. As antioxidants in nature, these two phytochemicals are beneficial to humans in preventing diseases, such as cancer, inflammation, and stroke [9]. A high level of total phenol and flavonoids in seedlings grown with a night temperature of 15 °C not only indicates a high value for humans, but also suggests benefits for the growth and development of plug seedlings to face abiotic and biotic stresses.

Photosynthesis is an important physiological process for biomass production in plants [48], and is regulated by temperature. The data showed that a night temperature of 15 °C up-regulated photosynthesis-related genes, including *GBSS*, *RBCL*, and *FDX*. In the photosynthetic chain, *FDX* codes the ferredoxin protein, which accepts and transfers electrons to the next receptor, NADP^+^ [28]. A higher expression of *FDX* with a night temperature of 15 °C implies an enhanced photosynthesis, resulting in higher biomass. In CO_2_ assimilation, *RBCL* plays a key role in CO_2_ fixation and regulates the photosynthesis rate. Up-regulated expression of *RBCL* suggested that a 15 °C night temperature promoted CO_2_ assimilation and synthesized more carbohydrates, which is consistent with the higher contents of soluble sugar and starch observed. In addition, expression of *GBSS* was positively modulated by the 15 °C night temperature, indicated by increased biomass and accumulation of starch. The lower expression of *GBSS*, *RBCL,* and *FDX* at night temperatures of 10 °C and 20 °C was possibly related to metabolic master regulators such as SnRKs which have specialized roles in controlling growth, stress responses, and photosynthesis program in plants [49,50]. More research is needed to clarify how SnRKs modulate photosynthetic-related gene expression, growth, and metabolism of plug seedlings under abiotic stress condition [50,51]. Another reason is perhaps due to H_2_O_2_ (Figure 3), since H_2_O_2_ has a negative effect on nucleic acids and proteins, leading to a degradation of photosynthetic genes and proteins [52]. Those results are in accordance with those of previous studies. For instance, Bukhov et al. [35] reported that a high temperature inhibited the ferredoxin-dependent electron transport in barley (*Hordeum vulgare* L.) leaves. Jin et al. [53] found that a high temperature decreased the activity of Rubisco and the quantum yield of photosystem (PS) II in *Euonymus japonicas* seedlings. Phan et al. [33] reported that a high temperature strongly inhibited the expression of *Granule-bound starch synthase* in grains during the early grain filling in rice (*O. sativa* L.).

The night temperature had an influence on the chlorophyll content. In this study, an elevated night temperature (15 °C) increased the chlorophyll content in both *A. membranaceus* and *C. lanceolata* plug seedlings (Figure 4A,B). The results were similar with others. Bertamini et al. [54] reported that a low night temperature significantly decreased the contents of photosynthetic pigments in grapevine “Lagrein”. In addition, the contents of chlorophyll and carotenoids were decreased with a low night temperature in the grapevine [55]. Moreover, an enhanced night temperature (6 °C higher than the control) decreased the chlorophyll content, quantum yield of PSII, and photosynthesis in *Sorghum bicolor* [56]. Lesjak and Calderini [23] also found a significant difference in the SPAD values between plants grown with an increased night temperature and the control. As a core pigment in the photosystem, chlorophyll is an essential component of light-harvesting complex for capturing photons and transferring energy to the reaction center of photosystems. Any abiotic or biotic stresses such as heat, chilling, and drought will degrade the chlorophyll content [57,58,59]. A greater content of chlorophyll in seedlings grown with a night temperature of 15 °C implies a higher photosynthetic efficiency, since a positive relationship between chlorophyll and photosynthesis had been proven [60]. This also partly explains the higher biomass and metabolites in seedlings grown with a night temperature of 15 °C.

Stomatal conductance was affected by the night temperature. In this study, the maximum stomatal conductance was found at 15 °C in both *A. membranaceus* and *C. lanceolata* plug seedlings. Similarly, Machado et al. [61] reported that the stomatal and mesophyll conductance in the leaves of orange plants decreased after diminishing the night temperature to 8 °C. Additionally, under a cool-night condition (20 °C), the stomatal conductance and the net CO_2_ uptake rate in *Phalaenopsis aphrodite* were significantly greater than those in the control (28 °C) [62]. In the current study, a greater stomatal conductance at a night temperature of 15°C implies a higher photosynthesis rate, which resulted in more primary and secondary metabolites such as soluble sugar, starch, total phenols, and flavonoids [63,64] (Figure 2).

## 4. Materials and Methods

### 4.1. Plant Materials and Treatments

*A. membranaceus* and *C. lanceolata* seeds were sown in 200-cell plug trays filled with the BioPlug Medium (FarmHannong Co. Ltd, Seoul, South Korea). After germination, uniform plug seedlings were selected and cultivated in three environmentally controlled chambers under the same day temperature (25 °C) and different night temperatures: 10 °C, 15 °C, or 20 °C. Each treatment was set up with the same relative humidity (75%), photoperiod (12 h), and light intensity (150 μmol·m^−2^·s^−1^ PPFD) provided by white LEDs. Before the experiment, the light intensity in all treatments was adjusted and calibrated using a photoradiometer (HD2102.1, Delta OHM, Padova, Italy). The plug seedlings were subirrigated once every two days with a multipurpose greenhouse nutrient solution (in mg·L^−1^ Ca(NO_3_)_2_·4H_2_O 737.0, KNO_3_ 343.4, KH_2_PO_4_ 163.2, K_2_SO_4_ 43.5, MgSO_4_·H_2_O 246.0, NH_4_NO_3_ 80.0, Fe-EDTA 15.0, H_3_BO_3_ 1.40, NaMoO_4_·2H_2_O 0.12, MnSO_4_·4H_2_O 2.10, and ZnSO_4_·7H_2_O 0.44 (pH 6.5 and electrical conductivity 1.5 mS·cm^−1^)) throughout the experiment. After four weeks of cultivation, some plug seedlings were harvested at 10:00 during the daytime for the measurement of the growth parameters, and others were frozen immediately in liquid nitrogen for further analyses. The Dickson’s quality index was calculated according to a previous formula [65]. The formula is

Dickson’s quality index = Total DW/((shoot length/stem diameter)+(shoot DW/root DW))(1)

where the DW means dry weight.

### 4.2. Contents of Soluble Sugar and Starch

For extracting the soluble sugar and starch, frozen leaves (0.2 g) were ground and then mixed with distilled water (14 mL) for 30 min at 100 °C. After centrifugation at 3000 rpm for 15 min, the supernatant was transferred into new tubes for the assay of soluble sugar. The residue was re-extracted in distilled water mixed with perchloric acid (2 mL, 52%) for the measurement of starch.

Contents of soluble sugar and starch were determined by the anthrone colorimetric method [66]. In summary, the supernatant (0.5 mL) was added to distilled water (1.9 mL) combined with anthrone (0.5 mL, 2%), and concentrated sulfuric acid (5 mL, 98%), followed by 15 min of incubation at 100 °C. The absorbance at 630 nm and 485 nm was recorded using a UV-spectrophotometer (Libra S22, Biochrom Ltd., Cambridge, UK), respectively. The calibration curves of soluble sugar and starch were made with standard solutions.

### 4.3. Contents of Total Phenols and Flavonoids

For extracting total phenols and flavonoids from frozen leaves, 80% methanol was prepared and used. The contents of total phenols and flavonoids were measured by the methods described by Manivannan et al. [67]. In brief, the extract solution (50 µL) was mixed with distilled water (900 µL), phenol reagent (500 µL, 1:1 water), and sodium carbonate (2.5%, 1 mL), followed by incubation for 40 min in dark. The absorbance at 765 nm was recorded. For the assay of total flavonoids, the extract solution (50 µL) was mixed with methyl alcohol (80%, 900 µL) and aluminium chloride (2%, 1 mL) and incubated for 30 min. The absorbance at 415 nm was recorded using a UV-spectrophotometer (Libra S22, Biochrom Ltd., Cambridge, UK). The total phenols and flavonoids were calculated from the standard gallic acid and quercetin calibration curve, respectively.

### 4.4. Hydrogen Peroxide Content

The hydrogen peroxide (H_2_O_2_) content in frozen leaves was assayed as previously done [66]. In detail, samples (100 mg) were ground and homogenized with the TCA solution (0.1%, 1.0 mL). After centrifugation for 15 min at 12,000 rpm, the supernatant was collected and transferred into a new tube. The supernatant (0.3 mL) was mixed with a sodium phosphate buffer (50 mM, 0.5 mL), and potassium iodide (1 M, 0.5 mL), followed by incubation for 30 min in the dark. The absorbance of the mixture at 395 nm was recorded using a UV spectrophotometer (Libra S22, Biochrom Ltd., Cambridge, UK).

### 4.5. Assessments of Chlorophyll Content and Stomatal Conductance

The chlorophyll content was determined before harvesting using the Plus Chlorophyll Meter (SPAD 502, Spectrum technologies, Korea). The stomatal conductance was measured on leaves at 10 am before harvesting using a Decagon Leaf Porometer SC-1 (Decagon Device Inc., Pullman, WA, USA), according to the introduction of this instrument.

### 4.6. Analysis of the Gene Expression by Quantitative Real-Time PCR

Leaf tissues of *A. membranaceus* and *C. lanceolata* were ground and homogenized in lysis buffer under an RNase-free condition. For RNA extraction and cDNA synthesis, a total RNA extraction kit (iNtRON Biotechnology, Seoul, Republic of Korea) and the PrimeScript RT Reagent Kit (Takara, Shiga, Japan) were used, according to manufacturer’s protocols. Total RNA was reverse transcribed to cDNA using the GoScript Reverse Transcription System (Promega, Madison, WI, USA). The gene expression was assayed using the Rotor-Gene Q detection system (Qiagen, Hilden, Germany), and was calculated using the 2^−∆∆Ct^ method [68]. All primers used in this study were designed by using Premier 5.0 (Premier Biosoft Inc., Palo Alto, CA, USA) and were shown in Table 3.

### 4.7. Data Collection and Analysis

The data were collected with three individual biological repeats and presented as the mean ± standard error. One-way analysis of variance (One-way ANOVA) was performed to estimate the differences among treatments, followed by Duncan’s multiple range test (*p* < 0.05) using SPSS (Statistical Package for the Social Sciences, version 21). All figures in this study were prepared using the OriginPro software (version 9.0).

## 5. Conclusions

In this study, the effects of the night temperature on the growth and development of plug seedling of *A. membranaceus* and *C. lanceolata* were demonstrated. A night temperature of 15 °C greatly upgraded the quality of plugs seedlings in both species, with a higher dry weight (shoot, root, and leaf), stem diameter, and DQI. Moreover, high and low night temperatures had a negative effect on primary and secondary metabolites, and a night temperature of 15 °C significantly increased the contents of soluble sugar, starch, total phenols, and flavonoids in *A. membranaceus* and *C. lanceolata*. Furthermore, the stomatal conductance and chlorophyll content were improved by a night temperature of 15 °C and decreased with night temperatures of 10 °C and 20 °C. An analysis of the gene expression showed that a 15 °C night temperature enhanced photosynthesis by up-regulating the expression of the *GBSS*, *RBCL*, and *FDX* genes. In conclusion, the results suggested that a night temperature of 15 °C is recommended for producing high quality plug seedlings of *A. membranaceus* and *C. lanceolata*. Many further studies should be carried out in the future, such as how night temperature affects carbohydrates allocation and partition from source to sink, how night temperature influences the expression of genes related to secondary metabolite biosynthesis and degradation, and how night temperature impacts the absorption and transport of mineral elements.

## Figures and Tables

**Table 1:** Effect of night temperature on the growth parameters of *A. membranaceus* plug seedlings.

Night Temperature (°C)	Length (cm)		Dry Weight (mg)		Leaf Area (cm^−2^)	Stem Diameter (mm)	Root DW: Shoot DW Ratio	Shoot Dry Weight per Shoot Length (g·m^−1^)	Root Dry Weight per Root Length (g·m^−1^)	Dickson’s Quality Index (×10^−4^)
	Shoot	Root	Shoot	Root						
10	10.8 ± 0.4 b ^z^	4.1 ± 0.3 b	54.9 ± 7.8 b	8.7 ± 1.2 b	4.3 ± 0.2 a	0.83 ± 0.03 b	0.16 ± 0.01 b	0.51 ± 0.07	0.21 ± 0.03 b	4.6 ± 0.6 b
15	14.0 ± 0.4 a	4.5 ± 0.5 b	78.2 ± 4.3 a	17.8 ± 1.7 a	4.8 ± 0.2 a	1.06 ± 0.04 a	0.23 ± 0.02 a	0.56 ± 0.03	0.42 ± 0.06 a	7.1 ± 0.4 a
20	13.4 ± 0.4 a	5.9 ± 0.2 a	72.8 ± 5.0 a	14.8 ± 1.3 a	3.5 ± 0.2 b	0.98 ± 0.03 a	0.20 ± 0.01 a	0.54 ± 0.03	0.25 ± 0.03 b	6.2 ± 0.5 a
F-test	***	**	*	**	**	**	**	NS	**	*

^z^ Different letters (a and b) indicate the significant separation within columns by Duncan’s multiple range test at a 0.05 level. NS, *, **, and ***, represent no significant or significant difference at *p* = 0.05, 0.01, or 0.001, respectively. Data were presented as the mean ± standard error (n = 6).

**Table 2:** Effect of night temperature on the growth parameters of *C. lanceolata* plug seedlings.

Night Temperature (°C)	Length (cm)		Dry Weight (mg)			Leaf Area (cm^−2^)	Stem Diameter (mm)	Root DW: Shoot DW Ratio	Shoot Dry Weight per Shoot Length (g·m^−1^)	Root Dry Weight per Root Length (g·m^−1^)	Dickson’s Quality Index (×10^−4^)
	Shoot	Root	Shoot	Root	Leaf						
10	15.1 ± 0.6 b ^z^	3.3 ± 0.3 b	33.4 ± 2.6 b	4.1 ± 0.4 b	7.1 ± 0.9 b	6.6 ± 0.6	1.46 ± 0.05 b	0.19 ± 0.02 b	0.22 ± 0.02	0.13 ± 0.02 b	3.4 ± 0.3 b
15	17.5 ± 0.7 a	3.9 ± 0.1 b	57.6 ± 3.9 a	9.0 ± 0.6 a	11.7 ± 0.8 a	7.7 ± 0.6	1.81 ± 0.05 a	0.29 ± 0.02 a	0.34 ± 0.03	0.23 ± 0.02 a	6.5 ± 0.5 a
20	17.8 ± 0.7 a	4.9 ± 0.3 a	51.0 ± 5.6 a	6.9 ± 1.3 a	9.4 ± 1.0 ab	8.0 ± 0.5	1.80 ± 0.04 a	0.23 ± 0.01 b	0.29 ± 0.04	0.15 ± 0.03 b	5.5 ± 0.8 a
F-test	*	**	**	***	**	NS	***	**	NS	**	**

^z^ Different letters (a and b) indicate significant separation within columns by Duncan’s multiple range test at a 0.05 level. NS, *, **, and ***, represent no significant or significant difference at *p* = 0.05, 0.01, or 0.001, respectively. Data were presented as the mean ± standard error (n = 6).

**Table 3:** The primer sequence used in this study to measure the gene expression using quantitative real-time PCR.

Gene	Forward Primer	Reverse Primer
*18S*	5′-CTCAACCATAAA CGATGCCGACC-3′	5′-AGTTTCAGCCTTGCGACCATACTCC-3′
*AmGBSS*	5′-ATAACATAGCGTATCAGGG-3′	5′-CTCGGTCAGATTCTAACACT-3′
*AmRBCL*	5′-TGGCTGTTCCTATCGTCA-3′	5′-AAGTAATCTCCCTTTCTCCT-3′
*β-Actin*	5′-CGAGAAGAGCTACGAGCTACCCGATGG-3′	5′-CTCGGTGCTAGGGCAGTGATCTCTTTGCT-3′
*ClFDX*	5′-CTTCGGCGTTTCTTCGT-3′	5′-CTGCCAAACCCTTGATAACT-3′
*ClRBCL*	5′-GCTTACCCATTAGACCTTT-3′	5′-GGGACGACCATACTTGTT-3′

*Am*, *Astragalus membranaceus*; *GBSS*, *granule-bound starch synthase*; *RBCL*, *ribulose bisphosphate carboxylase large chain*; *Cl*, *Codonopsis lanceolata*; and *FDX*, *ferredoxin*. *18S* and *β-Actin* were used as the housekeeping genes in *A. membranaceus* and *C. lanceolata*, respectively.
